# Illegal drug use is associated with poorer life satisfaction and self-rated health (SRH) in young people

**DOI:** 10.3389/fpsyt.2023.955626

**Published:** 2023-02-21

**Authors:** Weixi Kang

**Affiliations:** Imperial College London, London, United Kingdom

**Keywords:** illegal drug, self-rated health, life satisfaction, young people, substance use

## Abstract

Illegal drugs can bring negative health and psychological health consequences to people who use them. However, much less is known about illegal drug use and its association with life satisfaction and self-rated health (SRH) in young people in the context of the United Kingdom, which is important because SRH and life satisfaction are associated with important outcomes including morbidity and mortality. By analyzing data from a nationally representative sample with 2,173 people who do not use drugs and 506 people who use illegal drugs aged between 16 and 22 (mean = 18.73 ± 1.61) years old from Understanding Society: the UK Household Longitudinal Study (UKHLS) using a train-and-test approach and one-sample *t*-tests, the current study found that illegal drug use is negatively associated with life satisfaction (t(505) = −5.95, *p* < 0.001, 95% CI [−0.58, −0.21], Cohen’s *d* = −0.26) but not with SRH. Intervention programs and campaigns should be developed to prevent people from using illegal drugs, which may then avoid the negative consequence of poor life satisfaction associated with illegal drug use.

## Introduction

Illegal drug use is one of the main contributors to the global burden of disease (1) that has adverse health and psychological consequences (2). The overall prevalence of illegal drug use in 2018–2019 ranges from 5.9 to 12% in the United Kingdom. However, this number was particularly high for young people, which ranges from 21 to 28% (3). Moreover, according to Allen and Laborde (2), “Illicit drug use and dependence are associated with an increased risk of mental disorders, road-traffic accidents, fatal overdoses, infections from unsafe injection practices (e.g., contraction of HIV), suicide and violence (1, 4), and illicit drug dependence accounted for 20 million (95% UI: 15.3, 25.4 million) disability-adjusted life years (DALYs) worldwide in 2010 (4).” Given the negative consequences associated with illegal drug use, there has been progress in trying to identify factors that relate to illegal drug use (2), including individual, interpersonal, community, and policy factors as pointed out by the social-ecological model. For instance, poor mental health, peer pressure, deprived family environment are factors of illegal drug use (5), which are also related to life satisfaction and SRH.

Self-rated health (SHR) refers to the subjective evaluation of one’s overall health whereas life satisfaction refers to how a person likes his or her life (6). It has been suggested that drug use is negatively related to life satisfaction (7). For instance, Zullig et al. (7) found that drug use is negatively related to SRH in American high school students. Boyas et al. (8) found that drug use is negatively related to SRH between Latino and non-Hispanic white adolescents aged between 12–17 in the United States. Moschion and Powdthavee (9) found that cannabis use is negatively associated with life satisfaction and the decrease in life satisfaction following the use of drugs persists 6 months to a year after initial use in homeless or at risk of being homeless participants in Australia. Illegal drug use may relate to life satisfaction and SRH through pathways such as poor physical and mental health (5), deprived socioeconomic status (10), and social stigma (11).

Thus, although there are some studies about how drug use is related to SHR and life satisfaction in young and middle adolescence [aged below 17; e.g., (7, 8)] and adults [e.g., (12)], much less is known about how drug use is associated with life satisfaction in young people in late adolescence and emerging adulthood, particularly in the United Kingdom. In late adolescence and emerging adulthood, individuals have to achieve autonomy from their guardians and will experience shifts in social roles and some normative expectations for their own behaviors. In this period of time, young people explore various aspects of their life including education, work, leisure interests, romance, and worldviews. The lifestyle factors associated with this period appear conducive to facilitating the development of addictions such as drugs [see (13) and (14) for reviews]. Moreover, newly found autonomy can lead to various mental health problems such as stress, anxiety, and depression, which in turn contribute to illegal drug use (15).

Thus, the aim of the current study is to investigate how illegal drug use is related to life satisfaction and SRH in young adults in the United Kingdom while controlling for demographics and other substance use behavior. The current study hypothesizes that drug use is negatively related to both life satisfaction and SRH.

## Materials and methods

### Data

Data were used from Understanding Society: the UK Household Longitudinal Study (UKHLS). This data has been shown to be nationally representative when compared with data from population censuses. Members from households recruited at the first round of data collection were interviewed face-to-face by trained interviewers or through a self-completion online survey. The current study used data in Wave 7, which was collected between 2017 and 2018 (16). All data collections have been approved by the University of Essex Ethics Committee. Participants received informed consent before participating. This dataset is quite representative as shown by comparing it with population censuses (17). The question that asks about illegal drug use was only administrated to people aged between 15 and 22 and participants with any missing variables of interest were removed. Thus, there were 506 participants who indicated that they have used illegal drugs in the past year with a mean age of 18.73 ± 1.61 years old and 262 (51.78%) males left after removing missing variables and 2,173 participants who indicated that they never used illegal drugs during the past year with a mean age of 18.37 ± 1.71 years old and 928 (42.71%) males. Descriptive statistics can be found in Table 1.

**TABLE 1:** Descriptive statistics of demographic characteristics, other substance use, life satisfaction, and SRH in non-illegal drug use and illegal drug use.

	Non-illegal drug use (*N* = 2173)		Illegal drug use (*N* = 506)	
	Mean	SD	Mean	SD
Age	18.37	1.71	18.73	1.61
Monthly income	1259.39	1354.53	729.52	4620.40
Life satisfaction	5.27	1.49	4.72	1.60
SRH	3.74	0.95	3.54	0.98
	**N**	**%**	**N**	**%**
**Sex**				
Male	928	42.71	262	51.78
Female	1245	57.29	244	48.22
**Highest educational qualification**				
Below college	2005	92.27	473	93.48
College	168	7.73	33	6.52
**Residence**				
Urban	1726	79.43	377	74.51
Rural	447	20.57	129	25.49
**Past 12 months alcoholic drink**				
Yes	1385	63.74	377	74.51
No	788	36.26	129	25.49
**Smoker**				
Yes	129	5.94	172	33.99
No	2044	94.06	334	66.01
**Used e-cigarettes**				
Yes	337	15.51	242	52.17
No	1836	84.49	264	47.83

### Measures

#### Illegal drug use

Illegal drug use was measured by the question “Since 1/[interview month] / [interview year–1], how many times have you used or taken any illegal drugs?” “[interview month]” represents the actual month that participants completed the questionnaires, which varied across individuals. “[interview year–1]” represents the actual month that participants completed the questionnaires minus 1 year. Together, these dates can effectively ask whether participants have taken illegal drugs the previous year despite they may complete these questionnaires at different time points. Participants who indicated that they have never used illegal drugs in that given year were considered as people who do not use illegal drugs whereas participants who have used illegal drugs during the past year were classified as people who use illegal drugs.

#### Life satisfaction

Life satisfaction was measured using the question “How dissatisfied or satisfied are you with… your life overall?” using a seven-point scale ranging from 1 (not satisfied at all) to 7 (completely satisfied). The scores of this measurement were treated as continuous. The results of single-item measures and multi-item measures such as the Satisfaction with Life Scale (SWLS) have been shown to be very similar (18).

#### SHR

Self-rated health was measured by the question “In general, would you say your health is…” using a five-point scale ranging from 1 (excellent) to 5 (very poor). SRH scores were reverse coded, so now one means very poor and five means excellent. The scores of this measurement were treated as continuous. The reliability of this single measurement of subjective health is good (19).

#### Control variables

Control variables include age, sex, monthly income, highest educational qualification, whether or not participants live in the urban area, and whether or not participants have drunk alcohol in the past year, whether or not participants consider themselves as smokers, and whether or not participants have smoked e-cigarettes.

### Analysis

A train-and-test approach was used to analyze current data. First, participants were grouped into two groups based on whether they used illegal drugs. Second, two generalized linear models were applied to people who did not use illegal drugs during the past year by taking control variables as predictors and life satisfaction and SRH as the predicted variable, respectively, with “fitlm()” function in MATLAB 2018a. Visually inspecting the residual plots of these models led to the conclusion that residuals are normally distributed, thus linear models are suitable in this case. Third, these models were used to predict scores that are expected if people who used illegal drugs were people who did not use illegal drugs in the past year by taking their control variables including age, sex, monthly income, highest educational qualification, whether or not participants live in the urban area, whether or not participants have drunk alcohol in the past year, whether or not participants consider themselves as smokers, and whether or not participants have smoked e-cigarettes as predictors. Finally, two one-sample *t*-tests were applied to see if there are significant differences between the scores that were expected to see if they did not use illegal drugs the past year and their actual life satisfaction and SRH scores. All analyses were conducted on a customized script on MATLAB 2018a.

## Results

Descriptive statistics can be found in Table 1. The overall prevalence of illegal drug use was 18.89%. Young people who used illegal drugs during the past year received less college-level education, tended to live in a rural area, were more likely to consume alcohol during the past year, and were more likely to be a smoker and have used e-cigarettes than people who did not use illegal drugs during the past year.

The current study found that there is a main effect of age [*F*(1,2164) = 5.09, *p* < 0.05], sex [*F*(1,2164) = 6.10, *p* < 0.05], and smoking status [*F*(1,2164) = 5.05, *p* < 0.05] on life satisfaction among people who did not use illegal drugs in the past year. However, the main effect of monthly income, highest educational qualification, residence, drinking status, and vaping status was not significant.

Moreover, there was a main effect of sex [*F*(1,2164) = 40.66, *p* < 0.001], residence [*F*(1,2164) = 11.05, *p* < 0.001], smoking status [*F*(1,2164) = 19.80, *p* < 0.001], and vaping status [*F*(1, 2164) = 8.86, *p* < 0.01] on SRH among people who did not use illegal drugs the past year. However, the main effect of age, monthly income, highest educational qualification, and drinking status on SRH was not significant. The parameter estimates of these general linear models can be found in Table 2.

**TABLE 2:** The estimates (*b*) of linear models trained based on demographic and other substance use predictors of people who did not use illegal drugs the past year.

	Life satisfaction		SRH	
	*b*	*p*	*b*	*p*
Age	-0.05	<0.05	-0.02	0.06
Sex	-0.16	<0.05	-0.26	<0.001
Monthly income	0.00	0.26	0.00	0.13
Highest educational qualification	0.09	0.49	0.06	0.44
Residence	0.16	0.05	0.17	<0.001
Drinking status	-0.07	0.35	-0.06	0.19
Smoking status	0.32	<0.05	0.40	<0.001
E-cigarette using status	0.18	0.06	0.18	<0.01
*R* ^2^	0.014		0.045	

**FIGURE 1:**
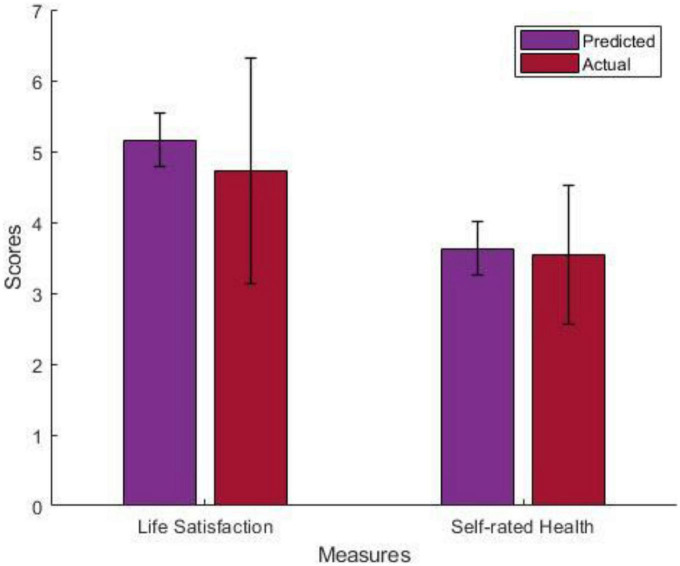
The mean and standard deviation of predicted and actual life satisfaction and SRH scores for people who used illegal drugs the past year.

The current study found that illegal drug use is negatively associated with life satisfaction (t(505) = −5.95, *p* < 0.001, 95% CI [−0.58, −0.21], Cohen’s *d* = −0.26) but not with SRH. The mean and standard deviation of predicted scores and actual scores for people who used illegal drugs in the past year were plotted in Figure 1.

## Discussion

The aim of the current study was to identify the relationship between illegal drug use and life satisfaction and SRH. By using a train-and-test approach and a *t*-test to analyze data from Understanding Society, the current study found that illegal drug use is negatively associated with life satisfaction but not SRH, which is largely consistent with the established literature regarding the association between substance use and poorer life satisfaction and SRH [e.g., (8, 12)]. Illegal drug use can bring not only negative consequences to physical but also psychological health. However, the current study did not find a relationship between drug use and SRH in young people, which may be explained by the fact that the current study controlled for other addictive behaviors including smoking, drinking, and vaping, which has been shown to be associated with poorer SRH [e.g., (7, 8)].

Risky behaviors in young people tend to cluster (20–23). Indeed, newly found autonomy in late adolescence and early adulthood can be a stressful time. In addition, this group of people is known to be less risk-aversive due to the perceptions of invincibility, which then leads to greater experimentation. Additionally, the underlying brain structures are not fully matured yet such as the inhibitory control network [see (24) for a review], which then leads to a higher chance of risky behavior. In addition, other factors such as uncertainty about the future, trauma, and mental health, may contribute to risky behavior such as illegal drug use, and poor life satisfaction.

It is reasonable to think that dissatisfaction with life among young people can be associated with other behaviors that are at risk such as sexual risk-taking, violence, aggression, suicide ideation, and dieting behaviors (7). Behavioral health specialists have begun to focus on the promotion of developmental assets in young people, including psychological wellbeing and adaptation (25–27), which broadens the scope of assessment in young people beyond traditionally assessed risk behaviors and psychiatric symptoms (23, 28, 29). Thus, these approaches may be sensitive to subtle, but clinically meaningful, changes in adolescent cognitive wellbeing. Life satisfaction measures can also extend the score of the wellbeing indicators in other large scale national survey in the United Kingdom.

Despite the strength of this study including well-controlled socioeconomic status and co-use of other substances including alcohol, cigarettes, and e-cigarette, there are some limitations. First, the current study is cross-sectional, which does not give support to strong causal inferences. Future studies should employ longitudinal approaches in order to establish causal association between illegal drug use and life satisfaction and SRH. Second, the current study is based on self-report measures, which can cause bias. Future studies should use more objective measurements to avoid these biases. Third, the type of illegal drug that participants used was not assessed. Future studies should investigate the various effects that different drugs may have on life satisfaction and SHR. Finally, illegal drug use is typically accompanied by psychiatric comorbidities and substance use disorders [e.g., (30, 31)]. However, they were not controlled in the model. Future studies should collect this information and control it to rule out their possible effects on life satisfaction and SRH.

To conclude, the current study investigated how life satisfaction and SRH are affected by illegal drug use status. By using an innovative train-and-test approach, the results showed that only life satisfaction but not SRH is affected by drug use status. There are also some implications that can be drawn from the current study. Interventions that help people quit illegal drug use and campaigns that prevent people from using illegal drugs are largely needed. Findings from the current study should also be utilized as a fact for educational purposes. Moreover, special attention may need to be given to young people who are at the stage of achieving autonomy, and who are at a higher risk of illegal drug use. For instance, providing consulting services at schools may provide a better chance for young people to successfully achieve autonomy and overcome difficulties in this period of time, which may reduce illegal drug use and promote life satisfaction.

## Data availability statement

Publicly available datasets were analyzed in this study. This data can be found here: https://www.understandingsociety.ac.uk.

## Ethics statement

The studies involving human participants were reviewed and approved by the University of Essex. The patients/participants provided their written informed consent to participate in this study.

## Author contributions

WK: conceptualization, data curation, formal analysis, investigation, methodology, resources, software, writing—original draft, and writing—review and editing.
